# Multiple Aneurysms or Pseudoaneurysms of the Gastroepiploic Artery: An Anecdotal Cause of Hemoperitoneum

**DOI:** 10.7759/cureus.56598

**Published:** 2024-03-20

**Authors:** Johan Sebastian Lopera-Valle, Brayan Muñoz-Caicedo, Julián Andrés Muñoz Durán

**Affiliations:** 1 Interventional Radiology, San Vicente Fundación, Medellín, COL; 2 Radiology, Universidad de Antioquia, Medellín, COL; 3 Interventional Radiology, Universidad de Antioquia, Medellin, COL

**Keywords:** gastroepiploic artery, aneurysm, pseudoaneurysm, hemoperitoneum, interventional radiology guided embolization, general and vascular surgery

## Abstract

Gastroepiploic artery aneurysms and pseudoaneurysms pose diagnostic challenges due to their rarity and overlapping radiological features. This case report presents an 82-year-old woman with sudden-onset severe abdominal pain with computed tomography revealing hemoperitoneum and saccular dilations adjacent to the stomach's greater curvature, suggestive of vascular pathology. Selective abdominal arteriography confirmed three saccular dilatations in the gastroepiploic artery, which were managed successfully with coil embolization. The discussion emphasizes the importance of accurate diagnosis, distinguishing between aneurysms and pseudoaneurysms, and prompt intervention to mitigate the risk of hemorrhagic complications of either of them. The case underscores the significance of endovascular management in such rare and critical scenarios.

## Introduction

Gastroepiploic artery aneurysms and pseudoaneurysms are rare [1]. The differentiation between the two entities is difficult and requires in radiological studies, apart from morphological criteria and contrast enhancement, that there be an etiology related to pseudoaneurysms. When there is none, the distinction is complex, and the risk of it being one or the other is 50% for each case, estimates based on retrospective studies and with few patients for this vascular bed [2]. When the diagnosis is pseudoaneurysm, the risk of hemorrhagic complications with fatal outcomes is high and not related to the size of the vascular lesion, so active interventional or surgical management is indicated.

The following is an illustrative and anecdotal case of multiple dilatations of the gastroepiploic artery as a cause of hemoperitoneum in an older woman without a pseudoaneurysm etiology, for which the probability of aneurysms or pseudoaneurysms is virtually equal. Its diagnostic and therapeutic approaches are described, ending with a brief literature review.

## Case presentation

An 82-year-old patient with a history of hypertension and dyslipidemia consulted the emergency department of a tertiary care hospital with sudden-onset and severe abdominal pain, predominantly in the mesogastrium. The pain was associated with pallor, sweating, and orthostatism. She denied fever, gastrointestinal bleeding, emesis, or changes in bowel habits.

Upon physical examination, she had a pulse of 108 beats per minute, blood pressure of 98/56 mmHg, and notable mucocutaneous pallor. Abdominal tenderness was elicited upon palpation, although no signs suggested peritoneal irritation. Laboratory results revealed a hemoglobin level of 9.2 g/dL as the only finding. Given the clinical presentation, a contrast-enhanced computed tomography (CT) of the abdomen was ordered to investigate potential surgical pathology.

**Figure 1:**
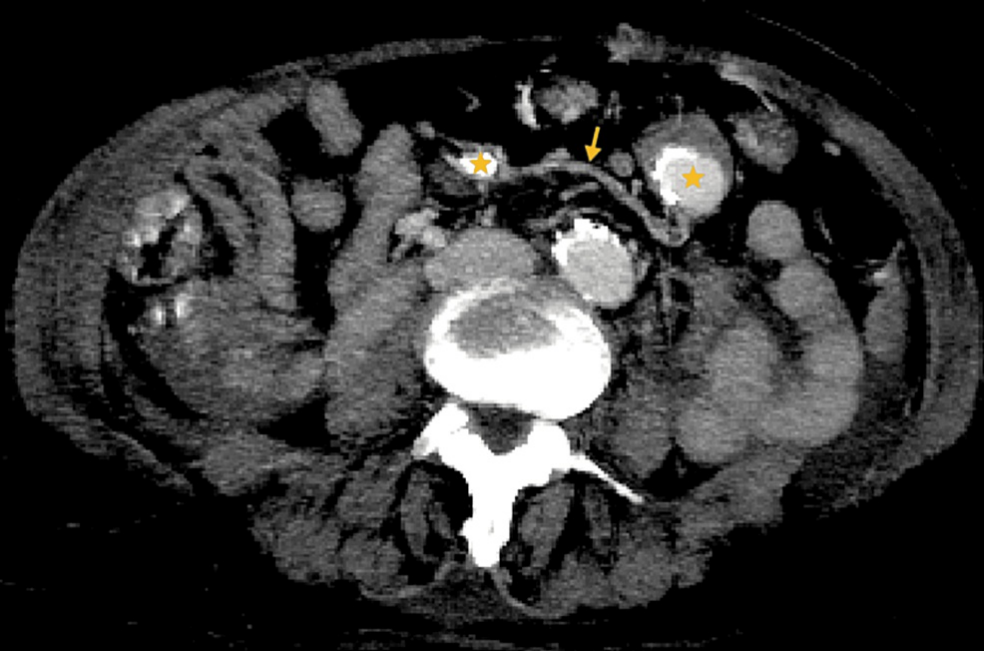
Abdominal CECT axial plane, in portal-venous phase and with positive-oral contrast administration. Yellow stars point to two saccular images with peripheral calcifications, close to the greater curvature of the stomach and in vascular trajectory (yellow arrow). CECT: Contrast-Enhanced Computed Tomography

The CT scan, performed with contrast administration in a single portal-venous phase with positive oral contrast, revealed two saccular images adjacent to the greater curvature of the stomach, exhibiting peripheral calcifications and measuring up to 28 x 23 mm, the largest of the two. These lesions appeared to follow a vascular trajectory. However, the absence of dedicated simple or arterial phases made interpretation challenging. Notably, the enhancement pattern of the vascular lesions with intravenous contrast resembled that of the oral contrast observed within adjacent small bowel loops (Figure 1).

**Figure 2:**
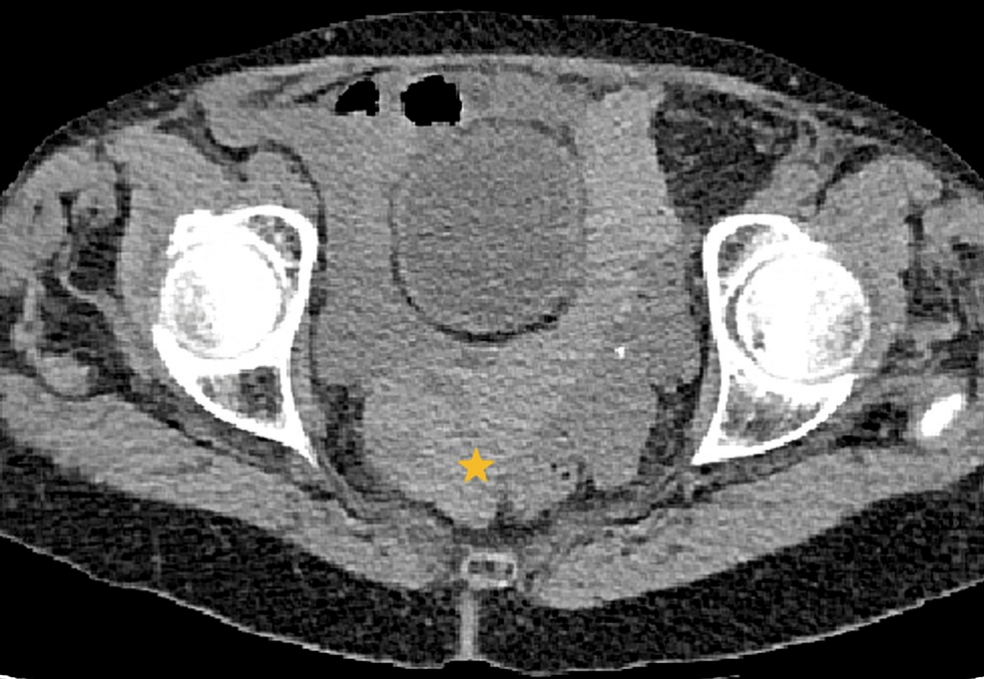
Abdominal CECT, portal venous phase, axial plane in the pelvis. The yellow star signals high-density free fluid in the pelvis (up to 86 HU), indicating hemoperitoneum.

High-density fluid was also observed in the pelvis (up to 86 Hounsfield Units -HU-), suggesting associated hemoperitoneum (Figure 2).

**Figure 3:**
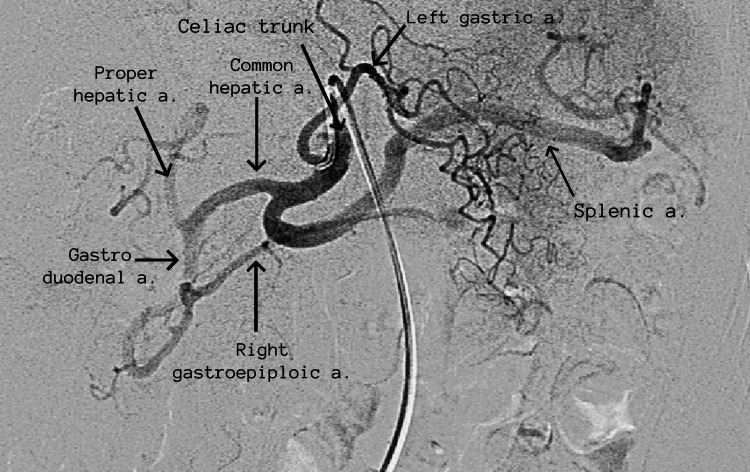
Selective abdominal arteriography image from the celiac trunk. The arteriography shows the usual vascular anatomy of the celiac trunk and its three main branches: splenic artery, left gastric, and common hepatic artery. Distal interest branches are labeled.

Selective abdominal arteriography was indicated with the diagnostic suspicion of visceral aneurysms as the cause of hemoperitoneum. Images of arteriography showed the usual vascular anatomy of the celiac trunk with its three main branches: splenic artery, left gastric artery, and common hepatic artery (Figure 3).

**Figure 4:**
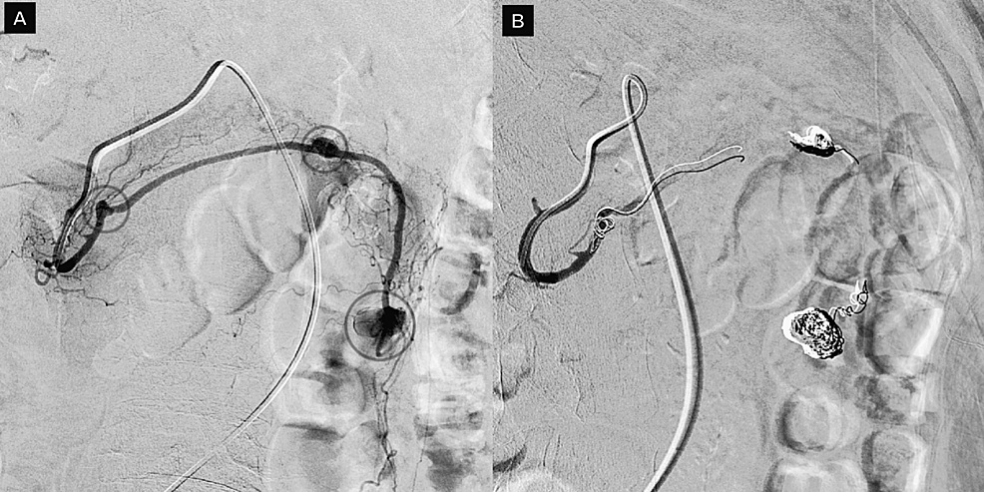
Selective arteriography from the gastroduodenal artery. (A) Three saccular dilatations in the gastroepiploic artery (circles). (B) Final arteriographic controls with the successful exclusion of gastroepiploic artery by coils implantation.

The catheter was navigated to the gastroduodenal artery, where new acquisitions were made. At this time, three saccular dilatations were documented in the gastroepiploic artery, the largest of them in the left gastroepiploic artery, one more in the middle third, and the smallest in the right gastroepiploic artery (Figure 4A). Using a microcatheter, occlusion of the described aneurysms was performed by implanting coils. Arteriographic controls showed successful exclusion of the gastroepiploic artery, with preservation of other visceral branches of the celiac trunk (Figure 4B).

After four days of hospital surveillance and ensuring clinical and laboratory stability, the patient was discharged without pain with recommendations and warning signs.

## Discussion

Gastroepiploic artery aneurysms (GEAA) are rare, accounting for approximately 3%-3.5% of visceral aneurysms [1,3]. These consist of focal dilation that affects all layers of the vascular wall. Atherosclerosis is the primary cause of GEAA, while less frequent causes encompass collagen disorders, degeneration of the middle arterial layer, and fibromuscular dysplasia [1].

On the other hand, pseudoaneurysms result from disrupting the intimal or middle layer of the artery with de-epithelialization of its wall surrounded by periarterial hematoma [1,4]. Given the rarity of GEAA [3], the estimation of true Gastroepiploic Artery Pseudoaneurysms (GEAP) has been performed retrospectively and, in a few cases, found that 50% of GEAA are actually GEAP [2]. Abdominal trauma with intimal injury and weakening of the vascular wall has been proposed as the main etiological factor of these pseudoaneurysms [5]. Other causes such as the enzymatic proteolytic effect in chronic pancreatitis, chronic cholecystitis, infectious causes (abscesses), iatrogenic surgical trauma or percutaneous biliary intervention, systemic vasculitis, segmental arterial mediolysis, and malignancy have been reported [4-7].

Patients are usually asymptomatic and are diagnosed as an incidental finding in imaging studies. Other patients suffer from some type of discomfort or epigastric abdominal pain. Those who are complicated by bleeding present with acute abdominal pain, anemia, and hypovolemic shock secondary to intraperitoneal hemorrhage [6,8-10]. Another reported complication is spontaneous fistulization of the colon [6].

Regarding diagnosis, CT angiography has sensitivity and specificity greater than 95% for detecting visceral artery aneurysms or pseudoaneurysms, requiring the arterial and venous phases since narrow-neck pseudoaneurysms can fill in this phase. Pseudoaneurysms need to be identified because they have implications for treatment. The diagnostic findings consist of morphological characteristics of a saccular vascular focal dilatation with irregular contour, eccentric location with eccentric thrombosis, density of behavior similar to the adjacent main artery in the different acquired phases and related in the imaging study with an etiology (e.g., trauma). If there is no clear related etiology for pseudoaneurysms, diagnosis is difficult [4], and the only way to adequately differentiate between pseudoaneurysms and aneurysms is pathologic [1]. The manipulation of rendering or projections of maximum intensity facilitates the identification of the finding and the vessel of origin. Similarly, the global anatomical representation in this study enables the identification of critical anatomical variants [11] and the complexity of the upcoming intervention.

Ultrasound can detect GEAP with the Yin-Yang sign to color Doppler. Magnetic resonance angiography is another option; however, it is limited by the decrease in contrast resolution, long acquisition times, lower availability, and higher costs. It may be an option in stable patients who tolerate the procedure and seek radiation limitation [4,5].

Angiography remains the Gold Standard; however, in the current scenario, it is only indicated when embolizing a pseudoaneurysm identified by the previous imaging methods is sought or when there is high clinical suspicion, but the non-invasive radiological studies are normal. An advantage of angiography is the ability to assess hemodynamics in real-time, which helps determine management [4,5].

Gastroepiploic aneurysms have a rupture and mortality risk of up to 90% and 70%, respectively, and therefore require management [1,4,12]. For pseudoaneurysms as such, there is no specific or high-quality literature. However, under the general principle for visceral pseudoaneurysms with an estimated risk of rupture of 2%-80% depending on the location and mortality of up to 100% and whose risk is not related to the size of the lesion, they should always be treated as soon as they are diagnosed [4].

In this regard, successful endovascular management of more than 90% makes it the main management option with embolization using coils, stents, plugs, liquid embolic agents, or thrombin injection [2-6,8,9,13]. Perforation of the lateral branch of the gastroduodenal artery during the interventional procedure has been reported [10]. When the endovascular pathway is complex due to vascular tortuosity with acute angles, percutaneous embolization with tomography or ultrasound guidance is another option [2,5,14]. Surgical management with laparotomies and hemorrhage control may be necessary, especially in cases where the distal circulation needs to be preserved or is at risk [1,13,15].

Finally, regarding the reported case, we know the limitation of the only portal phase acquired in the abdominal CT, even more so if we add it to the intestinal densities that could camouflage the vascular finding of the dilations. However, careful evaluation allowed the diagnosis to be made. Since no factor pointed to pseudoaneurysms as such, we decided to title the case report similarly to what Salehi et al. [10] did. Although the patient had atherosclerosis that could favor aneurysms, the presence of pseudoaneurysms cannot be ruled out. So, we leave the two options as diagnostic possibilities for better precision. The main finding of GEAA or GEAP is an anecdotal and unique case to our knowledge of the three dilations in this vascular territory with successful endovascular management that deserves publication.

## Conclusions

This case highlights the diagnostic complexity and therapeutic challenges of multiple GEAA or GEAP. Despite the rarity of these vascular abnormalities, prompt recognition and intervention are imperative to prevent and manage fatal hemorrhagic complications. The successful endovascular intervention, in this case, underscores the importance of employing a multidisciplinary approach involving radiological imaging and interventional techniques. Further studies and publication of similar cases are warranted to enhance understanding and management strategies for these uncommon but critical vascular pathologies.
